# Balancing Mobility and Driving Safety: Facilitators and Barriers for Driving Frequency Among Older Drivers

**DOI:** 10.1093/geroni/igaa057.1503

**Published:** 2020-12-16

**Authors:** Jiawei Cao, Jonathon Vivoda

**Affiliations:** Miami University, Oxford, Ohio, United States

## Abstract

Being able to drive safely ensures older adults’ transportation mobility and independence. It is also a key element for social participation and to achieve productive aging. However, when accounting for driving exposure, crash risk and crashed-related deaths increase for older drivers aged 70 years and plus. By analyzing data from NHATS, this study aimed to assess factors that might affect driving frequency (a five-category ordinal variable that measured how often people drove places). I examined whether productive aging activities (e.g., working, volunteering, caregiving, leisure, religious participation, civic engagement), perceived importance of social participation, mental wellbeing, physical capacity, cognition, sensory function, and community environment were either positively or negatively related to driving frequency. Age, gender, race, household size, marital status, and self-rated health were controlled for in this study. Results from ordinal regression showed that higher driving frequency was observed among older adults who reported that they worked for pay, provided care, visited friends and family, and went out for enjoyment. Higher driving frequency was also related to a higher perception of social participation importance. Inability to walk six blocks, lower scores on delayed word recall, and poorer vision were associated with lower driving frequency. Lower driving frequency was also found among women, married couples, and people with worth health and advanced age (80 years and older). These results suggest that higher driving frequency is associated with active social engagement and participation. New vehicle technologies or alternative transportation services and programs could be implemented to ensure safety, mobility, and social participation.

